# Engineering a Nanostructured Hybrid Gel System with Sodium Humate for Enhanced Wound Healing

**DOI:** 10.3390/jfb17040175

**Published:** 2026-04-01

**Authors:** Petya Peneva, Vesela Kokova, Elisaveta Apostolova, Plamen Simeonov, Nikolay Zahariev, Yana Gvozdeva, Dimitar Penkov, Rayna Hadjikinova, Ilia Bivolarski, Maria Koleva, Plamen Katsarov

**Affiliations:** 1 Department of Pharmaceutical Technology and Biopharmacy, Faculty of Pharmacy, Medical University of Plovdiv, Vasil Aprilov Str. 15A, 4002 Plovdiv, Bulgaria; petya.peneva@mu-plovdiv.bg (P.P.); plamen.simeonov@mu-plovdiv.bg (P.S.); nikolay.zahariev@mu-plovdiv.bg (N.Z.); yana.gvozdeva@mu-plovdiv.bg (Y.G.); dimitar.penkov@mu-plovdiv.bg (D.P.); 2 Department of Pharmacology, Toxicology and Pharmacotherapy, Faculty of Pharmacy, Medical University of Plovdiv, Vasil Aprilov Str. 15A, 4002 Plovdiv, Bulgaria; vesela.kokova@mu-plovdiv.bg (V.K.); elisaveta.apostolova@mu-plovdiv.bg (E.A.); 3 Research Institute at Medical University of Plovdiv (RIMU), 4002 Plovdiv, Bulgaria; 4 Department of Tobacco, Sugar, Vegetable and Essential Oils, University of Food Technologies, 4002 Plovdiv, Bulgaria; 5 Department of General and Clinical Pathology, Faculty of Medicine, Medical University of Plovdiv, 4000 Plovdiv, Bulgaria; iliya.bivolarski@mu-plovdiv.bg (I.B.); mariya.koleva@mu-plovdiv.bg (M.K.)

**Keywords:** hybrid gel, bigel, ultra-deformable vesicles, nanostructures, humic acid, wound healing

## Abstract

The development of effective topical drug delivery systems remains a key challenge in wound management, particularly for bioactive compounds with limited skin permeability. In this study, a nanostructured bigel system incorporating sodium humate-loaded ultra-deformable vesicles (UDVs) was developed and evaluated for wound healing applications. Sodium humate-loaded UDVs were prepared using a thin-layer hydration method, and the influence of key technological parameters (phospholipid/glycerol concentrations, sonication time) on vesicle size and encapsulation efficiency was investigated. An optimized UDV formulation characterized by small particle size, high stability, and high drug encapsulation efficiency was selected and incorporated into a bigel composed of hydroxypropyl methylcellulose hydrogel and andiroba oil oleogel. The developed bigels were characterized in terms of microstructure, physical stability, pH, spreadability, and rheological behavior, demonstrating suitable properties for dermal application. In vivo wound healing evaluation in rat wound models revealed that bigels containing sodium humate-loaded UDVs significantly enhanced wound closure and tissue regeneration compared to control and reference treatments. Histopathological analysis confirmed improved granulation tissue formation and complete epithelialization. Overall, the results demonstrate that the proposed UDV-loaded hybrid bigel represents a promising nanostructured platform for enhanced dermal delivery and wound healing therapy.

## 1. Introduction

Wound healing has been a current topic in caring for the well-being of the human body from ancient times to the present day. Wounds are defined as damage that compromises the integrity and structure of the skin, caused by external factors (cuts, burns, pressure), surgery, or pathological conditions such as diabetes or vascular diseases [1,2]. Wounds can be clinically divided into acute and chronic wounds. In acute wounds, the injury can be caused by a variety of factors, such as radiation, extreme temperature changes, or contact with chemicals. This type of wound heals spontaneously within 8–12 weeks [3,4]. Chronic wounds usually take longer to heal (even several months) due to continued inflammation. They can occur for several reasons, including tumors, infection, or physical agents [5]. Millions of people suffer from chronic wounds and large skin ulcers, which impair quality of life and pose major challenges for their families and healthcare systems worldwide [6,7,8]. In this regard, the efforts of researchers from various fields of science are aimed at creating different wound healing strategies.

Despite the extensive utilization of classical dermal delivery systems, considerable limitations persist. Conventional hydrogels, while providing a moist wound environment, often suffer from rapid moisture loss and possess a limited capacity to deliver large, complex therapeutic molecules (such as sodium humate) across biological barriers. Conversely, traditional ointments and emulsions have the potential to be excessively greasy, which can hinder patient compliance, or they are susceptible to thermodynamic instability [9,10].

Bigels are hybrid systems with a semi-solid consistency, obtained by mixing an oleogel and a hydrogel at a certain temperature [11,12]. These systems are a suitable platform for incorporating both hydrophilic and lipophilic active substances. Bigels are characterized by high stability due to their preparation from two pre-structured systems. They possess the characteristics of both gels, such as cooling, moisturizing and softening effects, easy spreading and easy washing from the skin [13,14]. It has been found that the combination of hydrogel and oleogel can lead to a synergistic effect of increasing the hydration of the *Stratum corneum* and the penetration of active substances through the skin, due to the presence of both an aqueous and an oily phase [15]. Based on all these described advantages, bigels are suitable for use alone or as carriers of active substances for skin treatment. The wound healing potential of bigel-based formulations has been well documented in the literature, with in vivo studies demonstrating enhanced tissue regeneration and superior healing outcomes compared to conventional formulations [16,17].

As part of hybrid bigel systems, the hydrogel phase plays a key role in ensuring biocompatibility, patient comfort, and effective delivery of bioactive compounds. Among the polymers commonly used for hydrogel preparation, hydroxypropyl methylcellulose (HPMC) is considered one of the most suitable materials for wound healing applications owing to its favorable physicochemical and biological characteristics. HPMC hydrogel is non-toxic, biocompatible and biodegradable, and has the ability to create a moist environment, which is essential for promoting cell migration, proliferation and differentiation [18,19]. Moreover, HPMC gel builds a physical barrier that protects the wound from further contamination [20,21]. Other advantages of this gel are related to the possibility of using it as a carrier of various substances with anti-inflammatory and antibacterial properties, its ability to stimulate angiogenesis, and to support the formation of granulation tissue, which is essential for wound closure and tissue regeneration [22,23]. Last but not least, the hydrogel can be easily applied and adapted to the shape of the wound and can be easily removed without further damaging the healing tissue [24,25].

As the second component of hybrid bigel systems, the oleogel phase provides an oil-structured matrix that enhances occlusivity, skin hydration, and delivery of lipophilic active substances. In this context, Andiroba oil was selected as the oil phase in the presented work due to its well-documented wound healing properties. Andiroba oil is extracted from the seeds of the fruit of the *Carapa guaianensis* tree, which traditionally grows in the humid tropical forests of the Amazon, Central America and Africa. Andiroba oil is a rich source of essential fatty acids such as oleic acid (±52%), palmitic acid (±28%), stearic acid (±10%) and linoleic acid (±11%), and other compounds such as steroids, triterpenes, mainly tetranortriterpenes or limonoids (2–5%), and vitamin E [26,27]. Due to this diverse chemical composition, the oil has a number of beneficial properties for the skin. It is believed to promote collagen synthesis, increase cell proliferation (fibroblasts, angiogenesis) and exhibit specific healing properties that can ensure proper wound healing with minimal scarring [28,29]. On the other hand, andiroba oil maintains hydration and reduces inflammation of the skin, which is of particular importance in the wound healing process [30]. Andiroba oil is considered a natural remedy that can relieve discomfort and pain in muscles and joints, including those caused by arthritis and rheumatism [31]. The wound healing efficacy of andiroba oil has been demonstrated in both in vitro and in vivo studies, showing significantly enhanced wound closure as well as improved epithelialization, fibroblast proliferation, and collagen deposition compared to controls [32,33].

While the bigel matrix itself contributes to wound healing, the incorporation of biologically active compounds can further potentiate its therapeutic effect through synergistic interactions. Currently, a variety of synthetic and semi-synthetic antibacterial agents are utilized in the management of wounds, including topical dyes (e.g., brilliant green), tissue-regenerating agents (e.g., methyluracil), and topical antibiotics. While these conventional treatments are often effective, they do have limitations. These limitations include tissue irritation, localized toxicity, potential for antimicrobial resistance, and undesirable tissue staining in the case of dyes [34]. Conseqently, a growing interest has recently been observed focusing on natural, accessible, and effective bioactive molecules for wound care applications. Among them, humic acid has emerged as a promising candidate [35]. Humic acid (sodium humate) is an organic polymer with a variety of biomedical applications, showing antioxidant, anti-inflammatory, and antimicrobial effects that are essential for the proper functioning of the complex wound healing process [36,37]. Sodium humate can significantly accelerate wound healing by reducing inflammatory cells such as neutrophils and eosinophils, promoting angiogenesis and stimulating fibroblast proliferation [38,39].

Despite its well-documented biological activity, the dermal delivery of humic acid is limited by its high molecular weight and complex, heterogeneous structure, which restrict diffusion through the stratum corneum [40,41]. These physicochemical characteristics may reduce its bioavailability at the wound site when applied in conventional topical formulations. Ultra-deformable vesicles (UDVs) are elastic lipid-based nanocarriers composed of phospholipids and edge activators, designed to undergo extensive deformation and pass through narrow intercellular spaces of the skin without losing structural integrity [42,43]. Incorporation of humic acid into UDVs is therefore considered a promising strategy to enhance its skin penetration, local bioavailability, and overall wound healing efficacy.

The aim of the present study was to develop and evaluate a novel nanostructured hybrid bigel system incorporating sodium humate-loaded ultra-deformable vesicles (UDVs) for enhanced dermal delivery and wound healing therapy. Specifically, the work focused on the formulation and optimization of sodium humate-loaded UDVs with small particle size and high encapsulation efficiency, followed by their incorporation into a hybrid bigel matrix designed to provide effective topical performance. The wound healing efficacy of the developed system was further assessed in vivo to demonstrate its therapeutic potential.

## 2. Materials and Methods

The following materials were used in this work: Methocel ^TM^ (Methocel K100M premium hydroxypropyl) (Colorcon Limited, Dartford, UK), cold-pressed *Carapa guaianensis* (Andiroba) Seed Oil, 100% pure base oil (manufactured by Alteya Organics LLC, Stara Zagora, Bulgaria). Humic acid sodium salt (sodium humate) and Span^®^ 60 were purchased from Sigma-Aldrich (St. Louis, MO, USA). Hydrogenated phosphatidylcholine (Lipoid H 100-3) was kindly gifted by Lipoid GmbH (Ludwigshafen, Germany). Zoletil and Meloxidyl were procured from Virbac’s (Virbac Corp., Carros, France) and ACCORD’s (ACCORD HEALTHCARE LIMITED, Devon, UK) regional distributors. Ethyl alcohol 70° 1 L (Chemax Pharma, Sofia, Bulgaria) as well as the standard product Herbal wonder^®^ (Bioherba, Plovdiv, Bulgaria) were acquired from a regional pharmacy. Eosin Y (1% aqueous solution, cat. No. 294/EOY-10-OT-2.5L), formaldehyde 4% (10% neutral buffer, cat. No. 294/FNB4-10L), Histanol 100 (cat. № 294/H100-5L), Histanol 95 (cat. No. 294/H95-5L), hematoxylin G3 (cat. No. 294/HEMG3-OT-2.5L), acetone (cat. No. 48/3413/5) and xylol (cat. No. 348/3410/20) were purchased from BIOCARE Medical (Pacheco, CA, USA). Flexible cohesive bandages (“b-flex”, 4.5 × 5 cm) were purchased from the importer (Toshev Farma Ltd., Shumen, Bulgaria, 60 Panayot Volov str.). All other reagents were of analytical grade

### 2.1. Preparation of Ultra-Deformable Vesicles (UDVs)

Ultra-deformable vesicles (UDVs) were prepared via thin-layer hydration method with slight modifications as described previously [44]. Briefly, phosphatidylcholine at varied concentrations was dissolved in a chloroform:methanol solution (2:1 *v*/*v* ratio) and the solvent was evaporated using a rotary vacuum evaporator BUCHI RII Rotavaport (BÜCHI Labortechnik AG, Flawil, Switzerland) under reduced pressure until a thin film was obtained. The film was kept under vacuum for the complete removal of the organic solvent. The film was then hydrated with a solution of PBS 7.4, glycerol and sodium humate for 2 h. Glycerol was used at different concentrations as a component of the UDVs that can act as an edge activator and vesicles stabilizer. After that the suspension was sonicated using a sonicator (Sonorex Bandelin electronic, Berlin, Germany) to optimize the size of the obtained vesicles. Furthermore, prior to inclusion in the bigel formulation, the UDVs were subjected to centrifugation at 18,000 rpm for 30 min at 4 °C using a Sigma 3-18KS centrifuge with a fixed-angle rotor (Sigma Laborzentrifugen GmbH, Osterode am Harz, Germany) to separate vesicles from any free drug.

### 2.2. Characterization of UDVs

The obtained vesicles with sodium humate were characterized in accordance with their average size and surface charge, which were determined by dynamic and electrophoretic light scattering methodologies employing the particle size analyzer Zetasizer UltraRed (Malvern Panalytical Ltd., Great Malvern, UK). All the measurements were carried out within 48 h after the preparation of the models and were conducted in triplicate. The entrapment efficiency of sodium humate in the obtained systems was determined indirectly. After separating the free drug from the UDVs by the aforementioned technique, the supernatant, containing non-incorporatedsodium humate, was analyzed with a UV-VIS spectrophotometer Evolution 300 (Thermo Fisher Scientific, Waltham, MA, USA) at wavelength 254 nm [45,46]. Entrapment efficiency (EE) was subsequently calculated based on Equation (1).

(1) $$EE\left(\%\right)=\frac{Total\;drug\;concentration-Total\;free\;drug\;concentration}{Total\;drug\;concentration}100$$

### 2.3. Preparation of Bigel Formulations

Bigel formulations were developed, which consist of hydrogel and oleogel phases in different ratios. The resulting complex structures are oleogel in hydrogel systems. The two gel phases were prepared separately, then mixed in different ratios.

The gelling agent Methocel ^TM^ (Methocel K100M premium hydroxypropyl) was used to prepare the hydrogel. To establish its optimal concentration, 3 hydrogel models were prepared, 2%, 2.5% and 3%, respectively. An accurately weighed amount of gelling agent was dispersed in purified water (drug-free bigels for control) or aqueous UDVs suspension, corresponding to the desired sodium humate concentration (sodium humate-loaded bigels) until it was completely wetted, then stirred with a stirrer (300 rpm), (Overhead Stirrer HS-100D, Witeg Labortechnic GmbH, Wertheim, Germany) for 20 min at room temperature (25 °C). The resulting hydrogel was left to stand for 24 h in order to remove the air included in it during stirring. The developed hydrogel models were visually evaluated for consistency and viscosity and the model with 2.5% gelling agent was selected for inclusion in the bigel.

The oleogel phase was prepared from Andiroba oil (*Carapa guaianensis*) and Span^®^60 (Sorbitan monostearate), with the gelling agent dissolved in the oil at 60 °C (Table 1). The bigel was obtained by adding the lipophilic phase heated to 60 °C to the hydrogel in portions under continuous stirring (300 rpm). After the entire amount of oleogel was incorporated, the stirring speed was increased to 700 rpm and the process continued for 30 min. Four biphasic compositions with different ratios of Methocel TM hydrogel and Andiroba oil oleogel were selected and developed. The obtained bigels were kept for two weeks to ensure the mutual distribution of the phases and to purge the system of air bubbles included in the preparation process. After this period of time, the bigels were visually inspected for color, odor, consistency and homogeneity.

For the purposes of the in vivo tests, two bigel models were designed with sodium humate concentrations of 1% *w*/*w*: model BGA20HA1 and 2% *w*/*w*: model BGA20HA2, and a bigel composition without drug was also formulated for control: model BGA20Control.

### 2.4. Characterization of Bigel Formulations

The prepared bigels were characterized through optical microscopy, centrifugation test, temperature stability test, pH, spreadability and rheological properties.

#### 2.4.1. Optical Microscopy

The microstructural analyses of the developed bigels were visualized using an optical bright field microscope Axio Scope A_1_ (Carl Zeiss Microscopy GmbH, Jena, Germany). One drop of each batch (~20 μL) was gently covered by a coverslip and the samples were observed at a magnification of 40× [47]. From each batch 50 randomly selected oleogel droplets were measured and, using Image J software 1.52a, the average droplet size and droplet size distribution were estimated.

#### 2.4.2. Centrifugation Test

This test was conducted to investigate the physical stability of the prepared bigels. For the purpose of the test, 5 g samples in tubes were centrifuged in a Cencom centrifuge (J.P. SELECTA, s.a., Abrera, Spain) at 3000 rpm for 30 min at a temperature of 25 °C. After the specified time, the bigels were visually assessed for any signs of phase separation.

#### 2.4.3. Temperature Stability Test

The thermal stability test was performed to assess the physical stability of two-phase systems under different temperature conditions. After being packaged in suitable containers, the model gels are stored at different temperatures, such as 4 °C (cold storage), 25 °C (room temperature) and 40 °C (accelerated stability). At certain time intervals (e.g., 7, 14 and 30 days) the deposited samples were examined for visible signs of physical instability—phase separation, change in consistency or separation of liquid from the hydrophilic or lipophilic phase. Based on the results of this test, the behavior of the bigel form under real and extreme conditions can be predicted [48].

#### 2.4.4. Determination of pH

The pH values of the obtained bigels were determined potentiometrically at room temperature. A calibrated pH meter (inoLab pH 720, WTW GmbH, Frankfurt am Main, Germany) was used. The electrode of the device was placed on the surface of the studied models and the result was read after one minute. The measurements were carried out three times for each of the models, with the pH being presented as the arithmetic mean ± standard deviation (SD) [49].

#### 2.4.5. Spreadability

The spreadability of the prepared bigels was evaluated by the parallel plate method as follows: 1 ± 0.01 g of sample was pressed between two horizontal glass plates (20 × 20 cm), where the upper plate weighed 125 ± 1 g. The experiment was carried out at room temperature, and the diameter of the resulting circle (Ø) was measured after 1 min. The analysis was performed three times and the results were presented as mean ± SD [50,51].

#### 2.4.6. Rheological Studies

The rheological properties of the samples were evaluated using a Rheotest 2 rheoviscosimeter (RHEOTEST, Medingen, Germany) at 25 °C over a shear-rate range of 0.17–72.9 s^−1^. The tangential stress (*τ*) and viscosity (*η*) values were subsequently calculated according to Equations (2) and (3).

(2) $$\tau =\frac{L\cdot Z}{10}$$

(3) $$\eta =\frac{\tau }{D}$$

where η is the viscosity, Pa·s; τ—tangential stress, Pa; D—velocity gradient, s^−1^; L—the readings read by the instrument indicator, sk·del.; Z—constant of the pair of coaxial measuring cylinders of the rheoviscosimeter.

### 2.5. In Vivo Wound Healing Analysis

#### 2.5.1. Animals and Treatment

Male Wistar rats weighing between 200 and 250 g were used in this study. The animals were maintained under standard laboratory conditions, including a temperature of 22 ± 1 °C, relative humidity of 45%, and a 12 h light/dark cycle, with free access to food and water. They were randomly divided into four groups, with eight rats in each group, and allowed to acclimate for seven days. Following wound induction, each rat was placed in a separate polyethylene cage measuring 48 cm × 35 cm × 20 cm (L × W × H).

The procedure for creating and treating the wounds was carried out in accordance with the method reported by Pereira Bessera et al. (2020) [52]. On the first experimental day, anesthesia was induced via intramuscular administration of Zoletil^®^ (tiletamine HCl + zolazepam) at a dose of 80 mg/kg. The dorsal fur of each rat was clipped, and the exposed skin was cleansed with 70° ethanol using cotton to minimize the risk of infection. Two wounds, each 8.0 mm in diameter, were then produced on the posterior dorsal area using a punch. After wound creation, the sites were disinfected again with 70° ethanol and photographed using a Nikon D3400 digital camera (Nikon Corp., Tokyo, Japan) at 55× magnification, maintaining a fixed distance of 26 cm between the camera and the wound surface. Subsequently, the wounds of each animal were treated in accordance with its assigned experimental group. The study assessed the following experimental groups: group 1—control—treated with bigel model BGA20Control; group 2—reference treatment—a standard product for wound healing (Herbal wonder^®^); group 3—bigel model BGA20HA1; group 4—bigel model BGA20HA2.

In groups 1, 3, and 4, both wounds on each animal were treated with the designated formulation, followed by placement of sterile cotton gauze over the applied gel. The dressing was secured using a self-adhesive, elastic, and waterproof bandage (“b-flex”, 4.5 cm × 5 m). The animals in group 2 were treated with Herbal Wonder^®^ ointment, which was applied to the wound area using a sterile cotton pad. The treated sites were then covered with cotton gauze and veterinary wrap bandages. To minimize postoperative pain and discomfort, all animals were administered a single oral dose of Meloxidyl^®^ (meloxicam) at a dose of 2 mg/kg via gastric gavage. Afterward, the rats were housed individually and observed for 24 h. Any animal exhibiting signs of pain or discomfort received an additional oral dose of Meloxidyl^®^ at a dose of 1 mg/kg.

The gel formulations or the reference product were readministered once daily in the morning for a total of 11 days. On experimental days 3, 5, 7, 9, and 11, wound photographs were taken prior to treatment application, following the previously described procedure. On day 11, the animals were euthanized by fracture of the spine in the neck area after receiving Xylazine at a dose of 5 mg/kg b.w. Subsequently, both wounds from each animal, including a 5 mm margin of surrounding tissue, were excised and processed for histopathological analysis.

#### 2.5.2. Determination of Wound Healing Percentage

The study protocol described by Kant et al. (2020) was used [53]. The macroscopic evaluation of the wound healing process was performed using digital photographs of the wounds. These photographs were taken on days 1, 3, 5, 7, 9, and 11. The measurement of the wound area was facilitated via image analysis software (ImageJ 1.52a, NIH). The percentage of wound healing was calculated by Equation (4):

(4) $$\mathrm{Wound}\;\mathrm{healing}\;(\%)=\left(\frac{A1-An}{A1}\right)\times 100$$

where A1 is the wound area on day 1 (the day of the surgery), and An is the wound area on the specific day.

#### 2.5.3. Histopathological Observation

The experimental protocol followed the methodology described by other authors [54]. The excised wounds were then fixed in a 10% neutral-buffered formalin solution and incorporated in paraffin blocks. The procedure of histological sample preparation has been previously documented [55]. The paraffin blocks were sectioned into 5 μm thin slices. Subsequently, the slices were subjected to hematoxylin and eosin staining. The samples were examined using Leica DM500 and Zeiss AXIO Scope A1 microscopes (Leica Microsystems, Wetzlar, Germany) by two independent pathologists who were unaware of the experimental groups. The evaluation of each sample was conducted according to the following scale, presented in Table 2 [54].

### 2.6. Statistical Analysis

UDV formulation data was statistically analyzed using Minitab LLC. Minitab^®^ Statistical Software, Version 21.1. (State College, PA, USA; 2023). The in vivo experimental data was analyzed with SPSS software, version 17.0 (IBM, Armonk, NY, USA). Results are presented as mean value ± standard deviation. Differences between group means were evaluated by one-way analysis of variance (ANOVA) and Tukey’s post hoc test. Statistical significance was reported when a *p*-value ≤ 0.05 was obtained.

## 3. Results

### 3.1. Preparation of Sodium Humate-Loaded Ultra-Deformable Vesicles (UDVs)

To enhance the topical delivery of sodium humate, as well as increase the wound healing potential, ultra-deformable vesicles were prepared. A 2^3^ + 1 full factorial design was conducted to determine the effect of the selected technological parameters (phospholipid concentration, glycerol concentration, sonication time) on the average size and entrapment efficiency of the prepared vesicles. Nine models were prepared and presented in Table 3 with the corresponding levels of variation for the selected factors.

After obtaining the results from the experiments, a factorial regression analysis was conducted using specialized statistical software Minitab 21.1, in order to determine the effect of the factors, as well as their interaction effects over the average size and entrapment efficiency. The ***ζ***-potential was excluded from the analysis as all of the obtained values were within the −40 mV range, which indicated that the prepared vesicles showed high stability, regardless of the variation levels of the factors.

**Figure 1:**
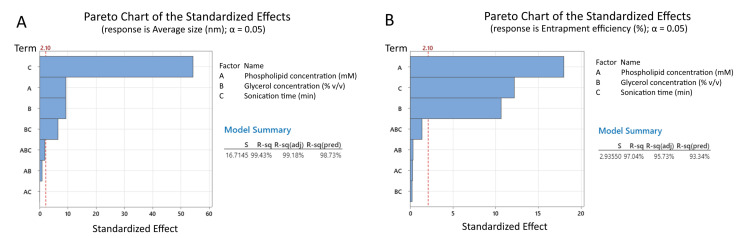
Pareto charts of the standardized effects and model summary on (**A**) average vesicle size and (**B**) entrapment efficiency of the formulated UDVs models.

The factorial regression analysis showed that the sonication time had the highest impact on the average vesicle size with a very high coefficient (−185.15), which was six times more than the effect of the other two parameters (Table 4). Phospholipid and glycerol concentrations, on the other hand, showed identical positive coefficients: +31.67 and +31.66, respectively. Overall, the model summary for the average vesicle size showed that the described model was highly accurate, showing that only 0.57% of the variations were due to random error or uncontrolled noise (R^2^ = 99.43%). The R^2^-adj value was also close to that of the R^2^, which signified the good correlation of the model (Figure 1A). The ANOVA analysis showed extremely high F-value (395.54) and *p* < 0.001, which shows that the model accurately identifies the highly significant main effects (*p* < 0.001) of all three variables, as well as the significant two-way interaction between glycerol concentration and sonication time (*p* < 0.001) (Table S1).

The model for the entrapment efficiency showed that, similar to the one for the average vesicle size, all three variables show a statistically significant effect (*p* < 0.001); however, in contrast to the other model, no statistically significant interactions were observed. The data showed that the factor with the most significant impact on the entrapment efficiency was the lipid concentration (+10.77). Glycerol was also found to increase the entrapment efficiency with the increase in its concentration; however, the magnitude was found to be lower (+6.37) than that of the phospholipid concentration. The sonication time also had a statistically significant effect on the entrapment efficiency, although the effect proved to be negative (−7.32). The model, evaluating entrapment efficiency, showed a high R^2^-value (97.04%), indicating that almost all variation can be explained. Apart from this, the R^2^-adj (95.73%) value was also found to be close to that of the R^2^, which signifies the good correlation of the model (Figure 1B). Analysis of variances (ANOVA) shows that the model is highly significant, demonstrating an F-value of 73.79, and *p* < 0.001 effectively identifies the highly significant main linear effects (*p* < 0.001) of the three individual variables, while indicating that their two-way and three-way interactions were not statistically significant (*p* > 0.05) (Table S1).

It should be noted that in both statistical models the central points (Ct Pt) showed no statistical significance (*p* > 0.05), which indicates that the described model shows a linear dependency between the varied parameters and the average size and the entrapment efficiency.

Based on the obtained results, model UDVs9 was selected for further incorporation into the bigel, as it provided a favorable balance between relatively small particle size and high encapsulation efficiency.

### 3.2. Preparation and Characterization of the Bigel Formulations

Biphasic gel compositions with different ratios of Methocel TM hydrogel/Andiroba oil oleogel were selected, based on literature data [57,58,59]. Four bigel models were obtained, coded BGA10, BGA20, BGA30 and BGA40, prepared at ratios of 90/10, 80/20, 70/30 and 60/40, respectively.

#### 3.2.1. Visual Appearance and Microstructural Analysis

**Figure 2:**
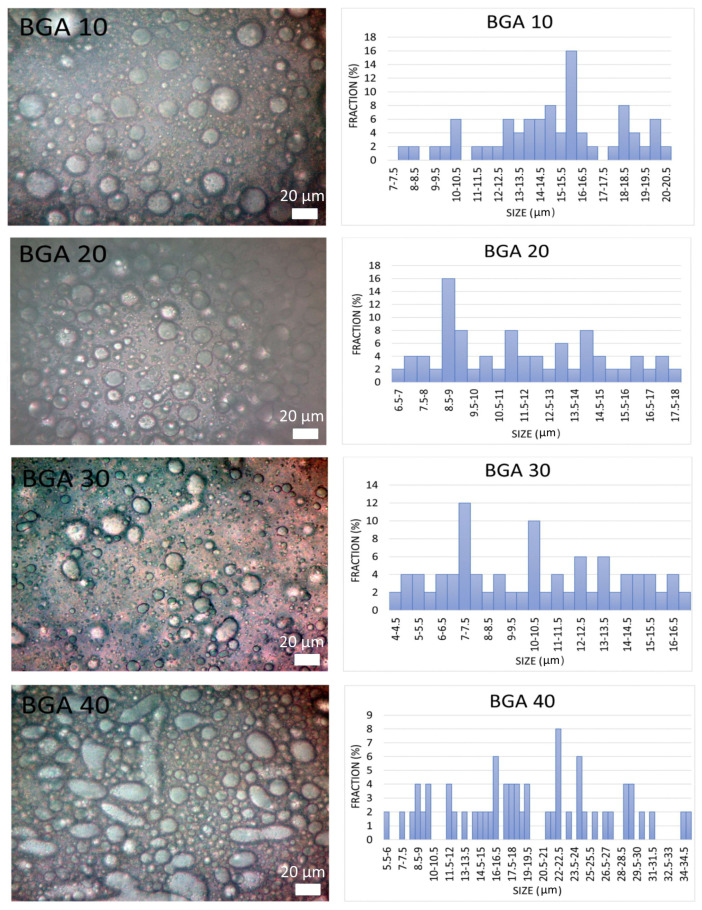
Photomicrographs and droplet size distribution of the bigel formulations.

The formulated bigel models had a creamy, smooth, homogeneous structure. In all samples, a faint aroma of andiroba oil was detected. The drug-free model used for control was pale yellow in color, while the models with sodium humate UDVs were brown, which was due to the natural color of the active substance. BGA10, BGA20 and BGA30 were not oily to the touch, unlike BGA40, in which oil droplets can be felt, although there was no phase separation. The results from the microscopic bigel microstructural analysis are presented in Figure 2.

The results showed that the batches prepared with 10%, 20% and 30% oleogel phase (BGA10, BGA20, BGA30) demonstrated a steady decrease in the mean droplet diameter, from 15.25 µm (BGA10) to 11.08 µm (BGA30) (Table 5).

The droplet size distributions for these three formulations were relatively narrow, ranging from 7.5 to 20.5 µm (BGA10), 6.5 to 18 µm (BGA20), and 4 to 17 µm (BGA30) (Table 5). In contrast, the formulation containing 40% oleogel (BGA40) showed a significant increase in both mean droplet diameter (19.37 µm) and size distribution range (5.5–34.5 µm).

#### 3.2.2. Stability and pH Evaluation

A centrifugation test was conducted to evaluate the physical stability of the bigels by their ability to maintain homogeneity after applying an external mechanical force. No phase separation was observed in all models, confirming their physical stability. Therefore, the amounts of oleogel used in the biphasic system from 10% to 40% ensured the physical stability of the hybrid system. Furthermore, the prepared bigels remained stable when stored at cold (4 °C), room (25 °C) and elevated (40 °C) temperatures, without signs of phase separation or liquid separation.

The pH values of the developed bigel models were within the range of the physiological pH of healthy skin (Table 6).

#### 3.2.3. Spreadability and Rheological Studies

The spreadability of the bigel formulations was determined by measuring the spreading diameter (Ø) when the models were pressed between two glass plates. The studied bigel models showed spreading diameters between 43.4 mm and 49.5 mm. The measurement data are summarized in Table 7.

**Figure 3:**
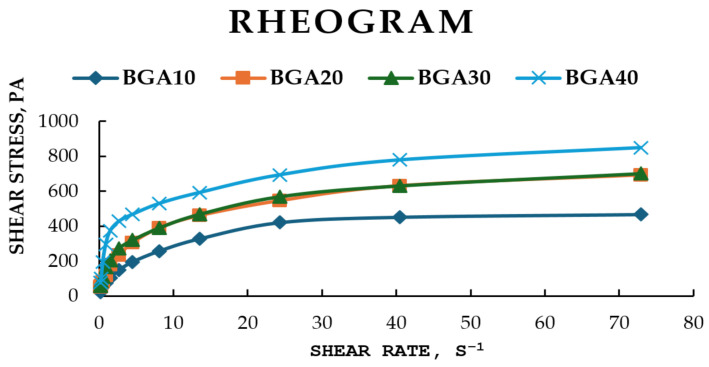
Rheograms at a temperature of 25 °C, sample type: BGA10, BGA20, BGA30, BGA40.

**Figure 4:**
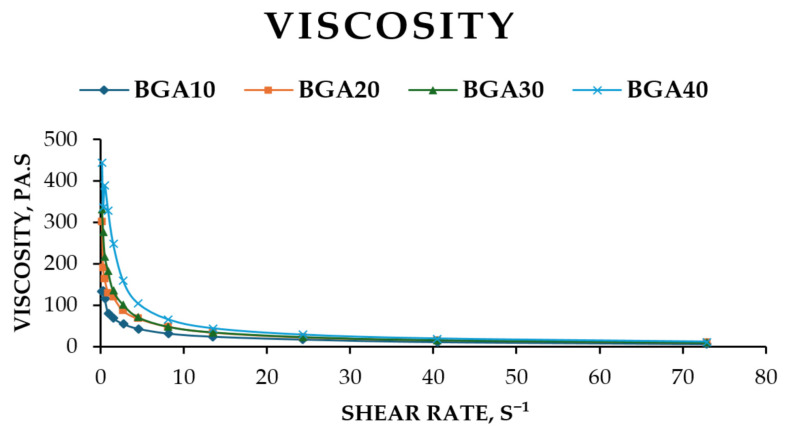
Viscosity of samples depending on the shear rate at a temperature of 25 °C, sample type: BGA10, BGA20, BGA30, BGA40.

The rheograms for the studied samples are presented in Figure 3. All formulated bigels exhibited non-Newtonian flow behavior. The lines of BGA20 and BGA30 almost overlap, which indicates identical behavior of the two models with increasing shear rate. Accordingly, between models BGA10 and BGA40, a greater difference in the increase in tangential stress is observed. This could be explained by the different amounts of hydrophobic phase in the bigel, 10% and 40%, respectively. Viscosity was selected as the primary rheological parameter for evaluation. The relationship between sample viscosity and shear rate is illustrated in Figure 4.

It is evident from Figure 4 that the relationship between viscosity and shear rate confirms the non-Newtonian flow behavior of the samples. The data showed that with increasing shear rate, the viscosity of the samples decreases. For example, when the shear rate (D) was increased from 1.5 to 8.1 s^−1^, the viscosity of the BGA10 sample decreased by approximately 51%, that of BGA20 by 61%, that of BGA30 by 64% and that of BGA40 by 73%. Also, BGA10, BGA20 and BGA 30 showed similar viscosities, while BGA40 showed the highest viscosity. This could be explained by the higher amount of gelling agent for andiroba oil, namely sorbitan monostearate in BGA40.

**Figure 5:**
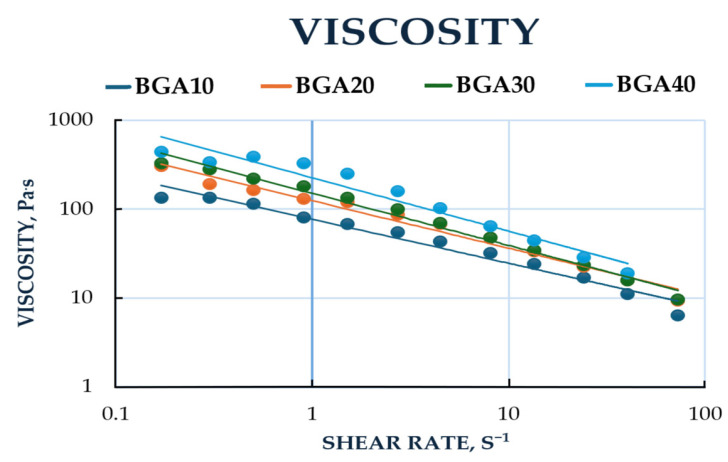
Viscosity of samples depending on the shear rate in logarithmic coordinates, sample type: BGA10, BGA20, BGA30, BGA40.

In order to quantitatively characterize the shear-thinning behavior observed in Figure 3, the experimental data were fitted to the Ostwald–de Waele (Power Law) model. The relationship between viscosity and shear rate plotted on logarithmic scales is presented in Figure 5. The figure shows that this relationship was linear in logarithmic coordinates and can be described using the Ostwald–de Waele power law for viscosity (Equation (5)) [60].

η = K·D^n−1^ (5)

where η is the viscosity of the sample, Pa·s; D is shear rate, s^−1^; K and n are coefficients.

The values of the coefficients in Equation (3), depending on the type of sample, are listed in Table 8.

R^2^ values indicate that the Ostwald–de Waele model is suitable for determining the viscosity of the samples.

The rheological parameters that were the focus of this study, including the consistency index (K) and the flow behavior index (n), are summarized in Table 8. For all hybrid gel formulations, the n values were found to be significantly less than 1 (ranging from 0.494 to 0.601), providing mathematical confirmation of their pseudoplastic nature. As the concentration of the nanostructured hybrid components increased from BGA10 to BGA40, the consistency index (K) increased from 76.81 to 225.55 Pa·s^n^. This trend suggests a progressive increase in the initial viscosity and structural density of the network, which can be attributed to the reinforcing effect of sodium humate within the polymer matrix. The flow behavior index (n) exhibited a modest upward trend in conjunction with an increase in concentration. This observation was made under the condition that all samples remained within the pseudoplastic region. This rheological profile is highly favorable for wound healing applications, as it ensures that the gel remains stable at rest (high K) but flows easily under shear (low n). This facilitates effortless application onto the wound site.

**Figure 6:**
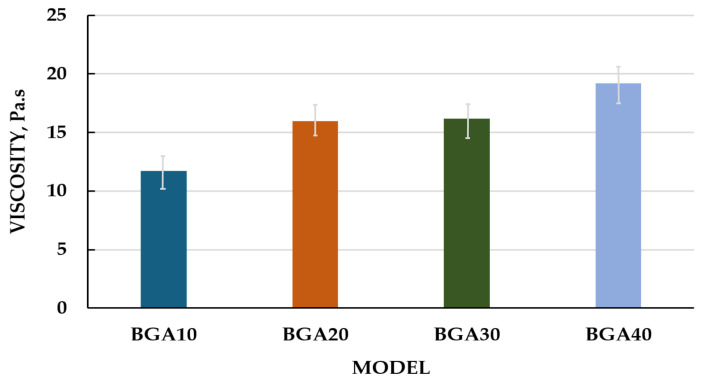
Dependence between viscosity and amount of oleogel, at D = 40.5 s^−1^.

The relationship between viscosity and the amount of oleogel in the formulation at a shear rate (D) of 40.5 s^−1^ is illustrated in Figure 6. The figure shows that increasing the oleogel content led to higher sample viscosities. Additionally, the absolute differences in viscosity between samples decrease as the oleogel content rises. For example, the viscosity difference between BGA10 and BGA20 is 4.28 Pa·s, between BGA20 and BGA30 is 0.2 Pa·s, and between BGA30 and BGA40 is 3.03 Pa·s.

Based on its overall characteristics, bigel model BGA20 was chosen for the subsequent in vivo experiments. While BGA20 and BGA30 exhibited similar optimal viscosities, spreading diameters, and droplet sizes, BGA20 was preferred because its higher hydrogel ratio (80:20) is hypothesized to create a more reliable film on the wound bed to maintain essential moisture and hydration. For the purposes of the in vivo tests, two bigel models were used with sodium humate concentrations of 1% *w*/*w*: model BGA20HA1 and 2% *w*/*w*: model BGA20HA2, and a bigel composition without drug was also formulated for control: model BGA20Control. From the perspective of pharmaceutical technology, concentrations of 1% and 2% were selected to establish an effective thermodynamic concentration gradient that would facilitate dermal permeation. Exceeding this concentration with a bulky, high-molecular-weight compound like sodium humate risks vesicle aggregation, loss of membrane deformability, and destabilization of the biphasic bigel network. Similar concentrations were also reported to be effective by other research groups [39,61].

### 3.3. In Vivo Evaluation of the Bigel Formulations

**Figure 7:**
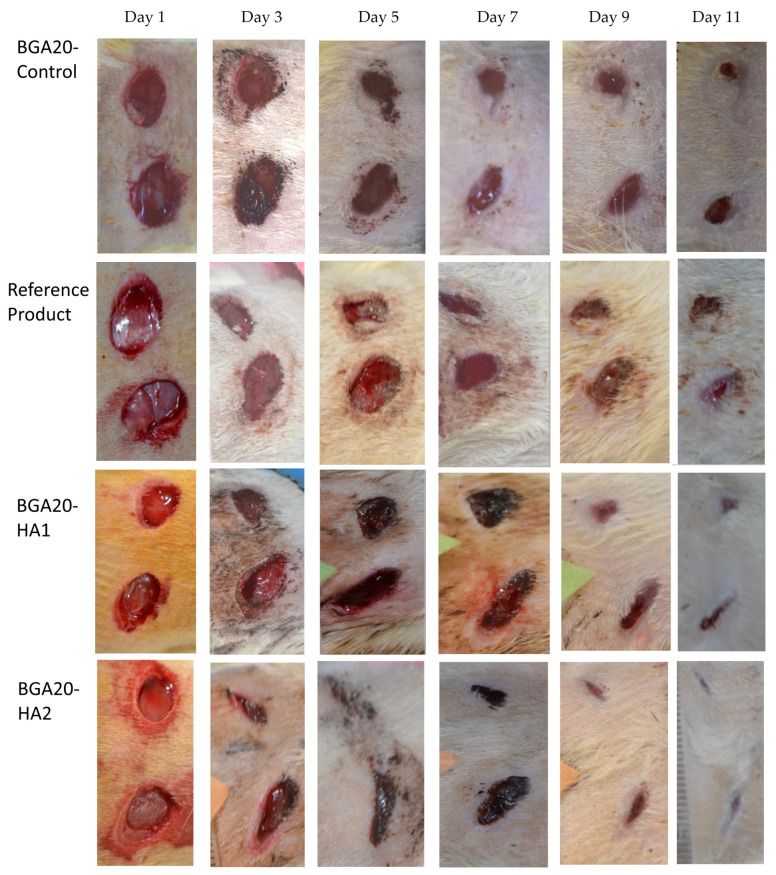
Macroscopic evaluation of wound contraction during treatment with bigel formulations without sodium humate (BGA20Control), the reference product (Herbal wonder^®^), bigels with sodium humate-loaded UDVs: sodium humate concentration in the bigel 1% (BGA20HA1), and 2% (BGA20HA2).

**Figure 8:**
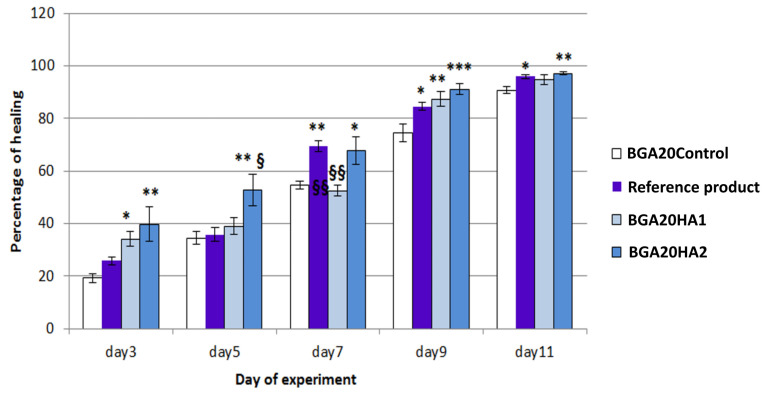
Changes in the percentage of wound healing after treatment with bigel formulations. Data are presented as mean ± SEM. The symbol * indicates *p* ≤ 0.05 vs. the control group on the same day; ** indicates *p* ≤ 0.01 vs. the control group on the same day; *** indicates *p* ≤ 0.001 vs. the control group on the same day; **^§^** indicates *p* ≤ 0.05 vs. the reference product group on the same day; **^§§^** indicates *p* ≤ 0.01 vs. the reference product group on the same day.

The in vivo wound healing performance of the developed bigel formulations was investigated in rats with experimentally induced dorsal skin wounds. Wound repair was monitored by macroscopic evaluation of wound contraction over an 11-day treatment period. Additionally, histopathological analysis of excised wound tissues was carried out on day 11 to assess granulation tissue formation, epithelialization, and overall tissue regeneration across the experimental groups. Macroscopic wound contraction was monitored during the course of treatment on days 1, 3, 5, 7, 9, and 11 (Figure 7). The reference product available on the market showed a wound healing effect mainly in the late phase of wound healing (7–11 days). The bigels with the lower sodium humate concentration (BGA20HA1) showed a statistically significant epithelializing effect compared to the control group on days 3 and 9, while for the bigels with the higher concentration of sodium humate (BGA20HA2), this effect was present throughout the entire experiment (Figure 8). On day 5 after wound induction, the wound healing effect was more pronounced for BGA20HA2 compared even to the reference product. On the 11th day, statistically significant differences compared to the control were observed in the groups treated with BGA20HA2 and the reference product. In conclusion, the bigels containing a higher concentration of sodium humate had a more pronounced wound healing effect, which was maintained during all stages of epithelialization.

Histopathological evaluation revealed clear differences among the experimental groups. In the control group treated with BGA20Control, the wound area showed an absence of granulation tissue formation as well as lack of epithelial coverage. In the group treated with the reference product, a moderately thickened granulation tissue was observed, composed mainly of inflammatory cells and fibroblasts, accompanied by pronounced neovascularization and moderate epithelial migration. Wounds treated with BGA20HA1 exhibited immature granulation tissue characterized by sparse inflammatory cells and a limited number of fibroblasts, along with minimal epithelial migration. In contrast, treatment with the BGA20HA2 bigel resulted in well-developed, thick granulation tissue predominantly composed of fibroblasts and collagen deposits, with complete epithelial coverage of the wound surface.

**Figure 9:**
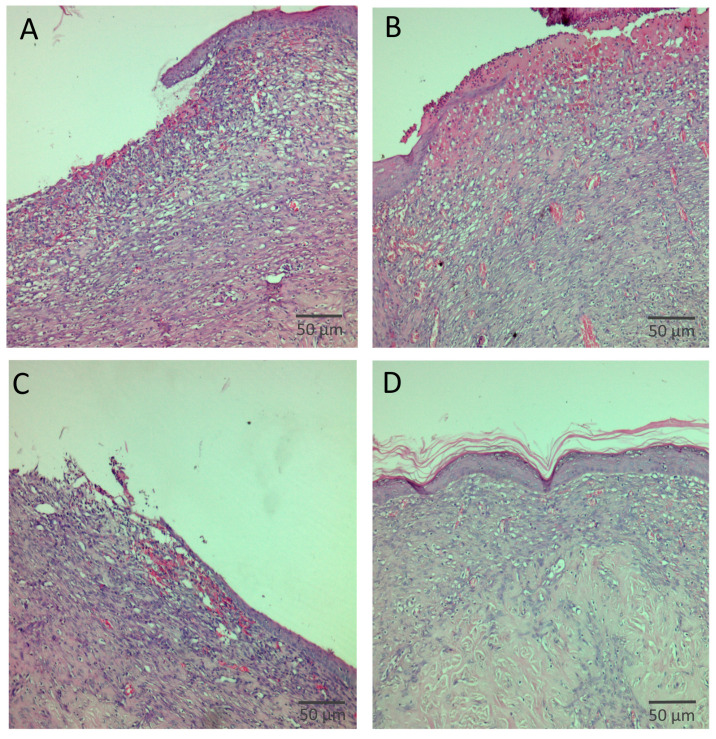
Histological examination of skin epithelization on day 11: Panel (**A**)—treatment with BGA20Control.; panel (**B**)—treatment with reference product; panel (**C**)—treatment with BGA20HA1; panel (**D**)—treatment with BGA20HA1. Staining: hematoxylin and eosin; Magnification 400×.

Therefore, on day 11, wound specimens were subjected to histopathological examination to evaluate tissue regeneration at the microscopic level (Figure 9). The corresponding distribution of histopathological scores is summarized in Table 9.

## 4. Discussion

### 4.1. Assessment of Technological Variables and Optimization of UDV Formulations

The first objective of the present study was to develop sodium humate-loaded ultra-deformable vesicles (UDVs) in order to facilitate the dermal delivery of the active substance. To achieve this, the most critical technological variables identified in the literature—namely phospholipid concentration, glycerol concentration, and sonication time—were systematically varied to evaluate their influence on the physicochemical properties of the vesicular systems. This approach enabled the identification of an optimal technological regime and UDV formulation characterized by small vesicle size in the range of 150–350 nm, suitable for enhanced skin penetration, while maintaining high drug encapsulation efficiency. Similar results were demonstrated by other groups that formulated these kind of vesicles [62,63].

The results from the regression analysis indicated that the sonication time had the highest impact on the average size, which was expected as the ultrasound wave provides high shear force that reduces vesicles size significantly [64]. The other two parameters (phospholipid and glycerol concentrations) showed an effect that was nearly identical in magnitude. As a whole, both parameters showed positive coefficients, meaning that as their concentration is increased the average size also increases. An interesting interaction with a statistically significant effect (*p* < 0.001) was the interaction between glycerol concentration and sonication time. Its negative coefficient indicated that the simultaneous increase in the glycerol concentration and extension of the sonication time resulted in significantly smaller vesicles. While glycerol can increase the viscosity of the suspension, thus slowing the ultrasound waves, it also can make the membranes of the liposomes softer. This can make them easily disrupted by low energy and the increased viscosity makes it harder for the vesicles to coalesce back again, resulting in overall smaller vesicles [65].

In terms of entrapment efficiency, the factor with the most significant impact was the lipid concentration. This can be explained with the fact that the more lipids present results in more liposomes that are being formed, which can increase the amount of water phase that is “captured” by the bilayers, resulting in an overall increase in the entrapment efficiency [66].

From the conducted statistical analysis, it was evident that there was an opposition of the effects of the variables on the average size and entrapment efficiency. For example, increasing the lipid concentration and glycerol concentration resulted in an increase in the entrapment efficiency, but also increase in the average size. The opposite applies for the sonication time: increasing the time of sonication resulted in smaller vesicles but also in a significant decrease in the entrapment efficiency. Model UDVs9 was selected as an appropriate model for incorporation in the bigel formulation, as it showed an appropriate average size for topical delivery (361.26 nm) and high entrapment efficiency of sodium humate (59.90%). Other models showed either large average size, which could limit the topical delivery of sodium humate, or low entrapment efficiency.

### 4.2. Optimization and Characterization of the Bigel Formulation

Following the optimization of the sodium humate-loaded ultra-deformable vesicles, the next objective of the study was to develop and characterize an optimal hybrid bigel formulation suitable for dermal application. Particular emphasis was placed on achieving a physically stable biphasic system with appropriate microstructural, rheological, and application-related properties. The variation in the hydrogel-to-oleogel ratio allowed for the identification of a bigel composition combining favorable stability, spreadability, and rheological behavior, essential for wound healing performance and patient acceptance.

The microscopic structural analysis of the developed bigel formulations showed that the amount of the oleogel phase in the system significantly affects the droplet size and droplet size distribution. The reduction in droplet size with the increase in the olegel content in models BGA10, BGA20 and BGA30 suggests that moderate increases in the dispersed oleogel phase enhance the efficiency of dispersion within the HPMC hydrogel matrix, likely due to increased shear fragmentation during mixing and improved distribution of the oleogel under these conditions [67]. This gradual narrowing of the size range further supports the formation of a more homogeneously dispersed biphasic system. The combination of smaller mean droplet sizes and reduced polydispersity typically correlates with enhanced physical stability in bigel systems, as smaller droplets are less prone to gravitational separation and coalescence [68]. The presence of larger droplets and a broader size distribution in model BGA40 suggests a reduced ability of the hydrogel network to effectively stabilize the dispersed oleogel phase at higher loadings. This microstructural change may be due to insufficient continuous-phase volume to support uniform dispersion, increased viscosity differences between phases, or the beginning of droplet coalescence during mixing [69]. Such features are generally linked to decreased physical stability and a higher likelihood of phase separation. These findings show that the increase in the oleogel amounts up to 30% lead to the formation of more finely dispersed and uniform droplets across the hydrogel matrix.

The formulated bigels showed the ability to maintain homogeneity after applying an external mechanical force according to the performed centrifugation test. Similar results were reported by Kasparaviciene et al. in their study of biphasic systems with diclofenac sodium and camphor [70]. Furthermore, the performed temperature stability test indicated the correct formation of the gel network and the mutual distribution of the hydrophilic and lipophilic gel in the biphasic system. Based on the results of the test, the behavior of the bigel system could be predicted in the event of non-compliance with the manufacturer’s instructions regarding the storage temperature regime.

The pH value of the dermal forms is important for physiological tolerance and the absence of possible skin irritation, redness and rashes [71]. The pH values of the developed bigel models were within the range of the physiological pH of healthy skin. The skin is covered with the so-called acid mantle, which is a delicate, slightly acidic film (pH 4.5–6.5) and in order not to disrupt its functions, it is recommended to apply dermal forms with similar pH values [72].

The spreadability of dermal formulations is another important characteristic because it determines the uniform distribution on the skin, the exact dosage and the therapeutic effect, the method of application (easy or more difficult placement on the skin) and last but not least, the patient’s acceptance of the product [73]. According to the Lardy et al. classification [74], all formulated bigels can be defined as semi-solid, because the established spreading diameter was Ø < 50 mm. The results showed that with increasing the amount of oleogel in the biphasic system, the spreading diameter decreased, which can be associated with the increase in the viscosity of the samples.

The rheological characteristics of dermal bigels are essential for their ability to spread on the skin, the release process of the incorporated active substance and the patient’s acceptance of the use of these forms. The main rheological parameters used to assess the quality of semi-solid forms are viscosity, shear stress, and flow type. The results from the rheological study showed that with increasing shear rate, the tangential stress of all samples increased, i.e., all studied models exhibited non-Newtonian flow behavior. It could be noted that with increasing the amount of oleogel in a biphasic system, the tangential stress also increases with increasing shear rate. A larger amount of oleogel leads to the formation of a denser and stronger internal structure (gel network), which requires more force for deformation [75]. The stronger bigel that was formed resisted movement at higher speeds, which is a key characteristic of non-Newtonian fluids. From the results obtained, we can conclude that the prepared gels are characterized by non-Newtonian pseudoplastic flow (shear-thinning behavior). This means that when at rest they are stable and dense, but become thin and spread easily on the skin under pressure (when rubbed), which leads to better application, uniform distribution on the skin and delivery of active ingredients, a key characteristic for topical products [76,77].

Based on the results obtained from all experimental analyses, it can be concluded that the prepared bigels possess excellent characteristics for semi-solid formulations for dermal application. The model compositions show pH within the physiological tolerance range of the skin and demonstrate physical stability when applying external force (centrifugation) as well as at different temperature regimes. In rheological studies, the bigels show pseudoplastic flow (shear-thinning behavior). This is a desirable characteristic for dermal semi-solid forms, because they are associated with the uniform application of the product on the skin and, accordingly, an optimal therapeutic effect. From microscopic observations, it was clear that models BGA20 and BGA 30 possess very similar average particle diameters of the dispersed phase (oleogel phase). The indicated models also show approximately the same viscosity and similar spreading diameter. Of these two models, the BGA20 model was chosen for the subsequent in vivo experiments, given that it contains a larger amount of hydrogel, which would create a more reliable film on the wound and maintain hydration.

### 4.3. Wound Healing Assessment

The bigel containing a higher concentration of sodium humate demonstrated a wound healing effect during all stages of epithelialization in comparison to the reference product, which was more effective during the late stages of epithelialization. A search of the scientific literature revealed no studies reporting the effects of gels containing 2% sodium humate. However, there was evidence for improved skin epithelization after treatment with gels containing lower concentrations of sodium humate. Ji et al. (2016) reported improved wound healing on days 6, 9 and 14 in rats treated with a 1% sodium humate gel [61]. The authors suggested involvement of transforming growth factor-*β* (TGF-*β*)/Smad signaling pathway and increased hydroxyproline in the observed effect. TGF-β is primarily involved in the late phase of epithelialization, which may explain the effects observed for BGA20HA2 [39]. Samiee-Rad et al. reported accelerated wound healing in rats treated with a gel containing 0.5% humic acid, observing reduced proliferation of inflammatory cells, which may account for the improved wound healing during the early phase of epithelialization [39].

Wound repair is one of the most complex physiological processes in the human body and progresses through four interrelated phases: hemostasis, inflammation, proliferation, and tissue remodeling [78,79]. Evidence from previous studies suggests that the anti-inflammatory and antioxidant properties of sodium humate may contribute significantly to this process. In experimental rat models, sodium humate has been shown to promote wound healing by accelerating wound contraction, increasing hydroxyproline levels, and enhancing overall tissue regeneration [61].

## 5. Conclusions

In this study, a nanostructured hybrid bigel system incorporating sodium humate-loaded ultra-deformable vesicles (UDVs) was successfully developed and evaluated as a topical wound healing platform. Optimization of the vesicular formulation enabled the production of stable UDVs with small particle size and high drug encapsulation efficiency, suitable for enhanced dermal delivery. The resulting hybrid bigel formulations exhibited appropriate physicochemical and rheological properties for dermal application. In vivo evaluation in a rat excision wound model demonstrated improved wound healing compared with the control formulation and reference treatment, with the higher sodium humate concentration showing the most pronounced effect, including accelerated wound contraction, enhanced granulation tissue formation, and epithelialization. These findings indicate that UDV-loaded hybrid bigels represent a promising strategy for improving the topical delivery and therapeutic efficacy of bioactive compounds with limited skin permeability. Future studies should focus on long-term stability, detailed release and permeation mechanisms, dose optimization, and clinical evaluation to further establish the applicability of this system for wound care.

## Figures and Tables

**Table 1:** Composition of hydrogel and oleogel per 100 g (*w*/*w*).

Hydrophilic Phase (Hydrogel Phase)		Lipophilic Phase (Oleogel Phase)	
Content	Amount (g)	Content	Amount (g)
Methocel K100M premium hydroxypropyl	2.5	Andiroba oil (*Carapa guianensis* Aubletet)	84.0
Purified water/UDVs aqueous suspension	97.5	Span^®^60	16.0

**Table 2:** Scale for histopathological evaluation of the excised wounds. Adapted from [56].

Score	Evaluation
1–3	Indicates the absence or minimal presence of fibroblast accumulation, with no evidence of granulation tissue formation or epithelial migration.
4–6	Represents a thin, early granulation matrix which is primarily composed of inflammatory cells, with few fibroblasts, minimal capillary formation and collagen deposition, and only slight epithelial migration.
7–9	Indicates a moderately developed granulation tissue characterized by abundant inflammatory cells, an increased number of fibroblasts, more extensive collagen deposition, pronounced neovascularization, and minimal-to-moderate epithelial migration.
10–12	Represents a well-formed, highly vascular granulation tissue characterized by a substantial presence of fibroblasts and significant collagen deposition, along with epithelial coverage extending from partial to complete closure of the wound surface.

**Table 3:** Design matrix with the formulation variables and corresponding sodium humate-loaded ultra-deformable vesicles model (n = 3).

Model	Phospholipid Concentration		Glycerol Concentration		Sonication Time		Average Size	ζ-Potential	EE Sod. Humate
	Level	mM	Level	%, *v*/*v*	Level	min	nm ± SD	mV ± SD	% ± SD
UDVs1	−1	1.0	−1	10	−1	5	471.92 ± 12.58	−49.83 ± 0.27	47.80 ± 3.06
UDVs2	+1	10.0	−1	10	−1	5	516.79 ± 6.83	−46.28 ± 0.53	68.42 ± 3.01
UDVs3	−1	1.0	+1	30	−1	5	561.36 ± 32.25	−35.11 ± 0.04	58.97 ± 2.32
UDVs4	+1	10.0	+1	30	−1	5	643.35 ± 4.52	−40.48 ± 0.63	82.12 ± 2.60
UDVs5	−1	1.0	−1	10	+1	15	133.59 ± 6.23	−39.84 ± 0.18	31.50 ± 1.80
UDVs6	+1	10.0	−1	10	+1	15	203.88 ± 9.72	−42.65 ± 0.20	54.84 ± 1.68
UDVs7	−1	1.0	+1	30	+1	15	159.28 ± 11.60	−40.61 ± 0.81	46.68 ± 0.76
UDVs8	+1	10.0	+1	30	+1	15	215.46 ± 11.48	−46.83 ± 0.97	55.75 ± 2.71
UDVs9	0	5.5	0	20	0	10	361.26 ± 3.39	−45.13 ± 0.54	59.90 ± 2.68

**Table 4:** Coded coefficients, obtained from the factorial regression analysis, evaluating the influence of formulation variables on the average vesicle size and entrapment efficiency of the designed UDVs models.

Term	Influence over the Average Size						Influence over the Entrapment Efficiency					
	Effect	Coef	SE Coef	T-Value	*p*-Value	VIF	Effect	Coef	SE Coef	T-Value	*p*-Value	VIF
Constant		363.20	3.41	106.45	<0.001			57.009	0.599	95.14	<0.001	
Variable I	63.34	31.67	3.41	9.28	<0.001	1.00	21.547	10.773	0.599	17.98	<0.001	1.00
Variable II	63.32	31.66	3.41	9.28	<0.001	1.00	12.742	6.371	0.599	10.63	<0.001	1.00
Variable III	−370.30	−185.15	3.41	−54.27	<0.001	1.00	−14.637	−7.318	0.599	−12.21	<0.001	1.00
Variables I*II	5.75	2.88	3.41	0.84	0.410	1.00	−0.433	−0.217	0.599	−0.36	0.722	1.00
Variables I*III	−0.09	−0.05	3.41	−0.01	0.989	1.00	−0.342	−0.171	0.599	−0.29	0.779	1.00
Variables II*III	−44.68	−22.34	3.41	−6.55	<0.001	1.00	−0.303	0.152	0.599	0.25	0.803	1.00
Variables I*II*III	−12.81	−6.40	3.41	−1.88	0.077	1.00	−1.698	−0.849	0.599	−1.42	0.174	1.00
Ct Pt		−1.9	10.2	−0.19	0.852	1.00		2.89	1.80	1.61	0.126	1.00

Variable I (Phospholipid concentration (mM)); Variable II (Glycerol concentration (%*v*/*v*)); Variable III (Sonication time (min.)).

**Table 5:** Composition and average particle size of the bigel formulations.

Batch	Oleogel (%)	Mean Diameter ± SD (µm)	Droplet Size Range (µm)
BGA10	10	15.25 ± 3.69	7.5–20.5
BGA20	20	12.27 ± 3.41	6.5–18.0
BGA30	30	11.08 ± 4.33	4.0–17.0
BGA40	40	19.37 ± 7.29	5.5–34.5

**Table 6:** pH values of the prepared bigels.

	BGA10	BGA20	BGA30	BGA40
pH value	6.25 ± 0.01	5.96 ± 0.02	5.83 ± 0.02	5.78 ± 0.03

**Table 7:** Spreadability of the prepared bigels.

Formulation	Spreading Diameter, Ø (mm)
BGA10	49.5 ± 0.15
BGA20	47.1 ± 0.02
BGA30	46.7 ± 0.08
BGA40	43.4 ± 0.23

**Table 8:** Values of the coefficients K, n and coefficient of determination R^2^.

Sample	Coefficient Values		
	K	n	R^2^
BGA10	76.808	0.494	0.967
BGA20	124.69	0.535	0.977
BGA30	150.64	0.587	0.984
BGA40	225.55	0.601	0.948

**Table 9:** Distribution of the number of samples in the groups according to their respective histopathological score.

		Score		
Group	1–3	4–6	7–9	10–12
BGA20Control	2	6	-	-
Reference product	-	2	5	1
BGA20HA1	-	4	4	-
BGA20HA2	-	-	2	6

## Data Availability

The original contributions presented in the study are included in the article, further inquiries can be directed to the corresponding author.

## Supplementary Materials

The following supporting information can be downloaded at: https://www.mdpi.com/article/10.3390/jfb17040175/s1, Table S1. Analysis of variance for entrapment efficiency and average size (ANOVA).

## Abbreviations

The following abbreviations are used in this manuscript:

EE	Entrapment Efficiency
HPMC	Hydroxypropyl Methylcellulose
PBS	Phosphate Buffer Saline
SD	Standard Deviation
TGF-*β*	Transforming Growth Factor-*β*
UDVs	Ultra-deformable Vesicles
