# Supplementary material for: Engineering a Nanostructured Hybrid Gel System with Sodium Humate for Enhanced Wound Healing
Source: J Funct Biomater. 2026 Apr 1;17(4):175. doi: 10.3390/jfb17040175 (PMC13117710; doi:10.3390/jfb17040175)
Supplement: Supplementary file 1 [file jfb-17-00175-s001.zip › jfb-4191930-supplementary.pdf]

Supplementary materials

Engineering a Nanostructured Hybrid Gel System with Sodium Humate for Enhanced Wound Healing

Table S1. Analysis of variance for entrapment efficiency and average size (ANOVA).

**Analysis of Variance for Entrapment efficiency (%)**					
**Source**	**DF**	**Adj SS**	**Adj MS**	**F-Value**	**P-Value**
Model	8	492893	61612	129,91	0,000
Linear	3	413925	137975	290,92	0,000
Lipid concentration	1	292141	292141	615,97	0,000
PC:DOTAP	1	4786	4786	10,09	0,005
Lipid phase: Xanthohumol	1	116999	116999	246,69	0,000
2-Way Interactions	3	78847	26282	55,42	0,000
Lipid concentration*PC:DOTAP	1	2225	2225	4,69	0,044
Lipid concentration*Lipid phase: Xanthohumol	1	72677	72677	153,24	0,000
PC:DOTAP*Lipid phase: Xanthohumol	1	3945	3945	8,32	0,010
3-Way Interactions	1	3	3	0,01	0,942
Lipid concentration*PC:DOTAP*Lipid phase: Xanthohumol	1	3	3	0,01	0,942
Curvature	1	118	118	0,25	0,624
Error	18	8537	474		
Total	26	501430			
**Analysis of Variance for Averge size (nm)**					
**Source**	**DF**	**Adj SS**	**Adj MS**	**F-Value**	**P-Value**
Model	8	884034	110504	395.54	< 0.001
Linear	3	870861	290287	1039.06	< 0.001
Lipid concentration	1	24069	24069	86.15	< 0.001
PC:DOTAP	1	24056	24056	86.11	< 0.001
Lipid phase: Xanthohumol	1	822736	822736	2944.93	< 0.001
2-Way Interactions	3	12179	4060	14.53	< 0.001
Lipid concentration*PC:DOTAP	1	199	199	0.71	0.410
Lipid concentration*Lipid phase: Xanthohumol	1	0	0	0.00	0.989
PC:DOTAP*Lipid phase: Xanthohumol	1	11980	11980	42.88	< 0.001
3-Way Interactions	1	984	984	3.52	0.077
Lipid concentration*PC:DOTAP*Lipid phase: Xanthohumol	1	984	984	3.52	0.077
Curvature	1	10	10	0.04	0.852
Error	18	5029	279		
Total	26	889062			
